# Law, Responsibility, and the Brain

**DOI:** 10.1371/journal.pbio.0050103

**Published:** 2007-04-17

**Authors:** Dean Mobbs, Hakwan C Lau, Owen D Jones, Christopher D Frith

## Abstract

Brain-imaging studies have reinvigorated the neurophilosophical and legal debate of whether we are free agents in control of our own actions or mere prisoners of a biologically determined brain.

Archaeological discoveries of traumatic injuries in primitive hominid skulls strongly hint that our species has a long history of violence [1]. Despite repeated attempts throughout history, including efforts to eliminate violence through the imposition of criminal sanctions, we have yet to dispel our violent nature. Consequently, criminal violence remains a common feature of most societies. As policy-makers seek deeper understandings of criminally violent and anti-social behaviour, many contemporary neuroscientists assume that the essential ingredients of the human condition, including free will, empathy, and morality, are the calculable consequences of an immense assembly of neurons firing. Intuitively, this view opposes Cartesian dualism (i.e., the brain and mind are separate, but interacting, entities) and assumes that violence and antisocial behaviour emanate from a mechanistically determined brain (see Box 1).

**Box 1. Should We Rethink Free Will?**

Research linking the brain to antisocial and criminal behaviour also raises neurophilosophical questions concerning our liberty. Most neuroscientists hold that “minds are simply what brains do” [62]. Indeed, with the omission of metaphysical constructs like the “mind”, many take the view that we are tied to the physical brain and, as a consequence, have little personal choice. A series of classic, yet controversial, studies by Benjamin Libet and colleagues showed that brain activity associated with deliberate decisions can be detected shortly before we are conscious of making the decision [63]. In these studies, participants reported when they first felt the intention to make a spontaneous movement by noting the position of a dot moving on computer screen. They apparently first became aware of their intentions about 200 milliseconds before action execution, which is later than the onset of the so-called readiness potential (or “bereitschaftspotential”) recorded from the scalp prior to movement. Despite criticisms about the accuracy of this timing method, recent research [64,65] has shown that if anything, the actual onset of conscious intention is likely to be even later. Moreover, psychologists report that our attributions of agency to actions are often illusory [66].

Despite these claims, free will as a concept is still unlikely to be eliminated. Clearly free will is a prerequisite for moral agency, and for society to run smoothly, we all need to believe that we are in full control of our actions. Not surprisingly, some have tried to find a middle ground in this argument. For example, Raine has entertained the idea that free will should be viewed along a “dimension rather than a dichotomy” ([31], p. 320), while Gazzaniga has argued that “brains are automatic, but people are free” ([52], p. 98). Is it reasonable, however, to posit that some people are more free than others? For example, few can dispute the fact that brain diseases such as schizophrenia and Huntington disease reduce the ability to act freely. Nonetheless, most juries may never have explicitly discussed the concept of free will [52]. Neurophilosophy may play an important role in understanding and updating the intuitions concerning free will and responsibility that may implicitly underlie juror deliberations.

From this standpoint, the exciting discoveries of neuroscience resonate far beyond mere philosophical banter and may have important implications for the way government institutions, including education and legal systems, operate. For example, to the extent that legal systems attempt both to move behaviour in socially desirable directions and also to adjudicate transgressions fairly, the legal system's effectiveness can be improved by deepening our understandings about why people behave as they do and both how and why people respond to various changes in legal incentives. Specifically, neuroscience may have important implications for both how we understand the multiple influences on violent behaviour and how the legal system may better engage with violent criminals.

## Studies of the Prefrontal Cortex in Anti-Social and Violent Populations

The birth of what may be coined modern “forensic neurology” lies in John Harlow's 19th century observations of Phineas P. Gage [2]. Gage, a railroad worker, suffered the unfortunate experience of having an iron bar blasted through the front of his brain, which resulted in extensive damage to the prefrontal cortex (PFC). Despite Gage's miraculous physical and intellectual recovery, conspicuous changes in his personality were reported. Briefly, the once courteous and diligent man became explicitly anti-social. As Gage's friends famously articulated, “Gage is no longer Gage”. Since Harlow's lurid description, computerized reconstructions based on Gage's skull fractures have determined more precisely the damaged PFC regions, which current evidence associates with autonomic, social, and affect regulation [3]. The case of Phineas Gage is compelling to both neuroscientists and legal thinkers because it provided the first indication that reasoning and regard for others can be compromised by frontal lobe injury. Harlow's observations have led many experts to speculate that neurological insult may be a prominent factor in recidivistic and violent criminal transgressions.

Modern empirical endeavours support the claim that the human PFC, a latecomer in the brain's phylogenic history, is what makes us rational, intellectual, and moral entities (Table 1). For example, several studies on patients with focal frontal lobe injuries have supported Harlow's case [4,5]. In one of the largest studies of patients with brain damage to date, Grafman and colleagues found that increased aggressive/violent scale scores were most strongly associated with similarly localized PFC lesions in a sample of 279 veterans of the Vietnam War [6]. Higher scores were, however, mostly associated with verbal aggression and less so with physical aggression, again fitting with Harlow's observations of Gage [2]. These studies, along with clinical observations, have led many to suggest that damage to the PFC results in “acquired sociopathy” or “pseudopsychopathy”.

**Table 1:**
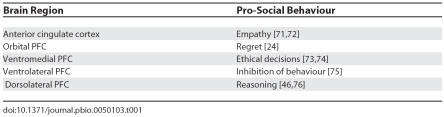
Examples of Prefrontal Brain Regions Associated with Pro-Social Behaviour

Given the PFC's historical and theoretical relevance to adaptive social behaviour, it is not surprising that this region was among the first to be examined in anti-social and violent populations. Raine and colleagues used noninvasive structural brain imaging to show an 11% reduction in PFC grey matter in patients with anti-social personality disorder (APD) [7]. These decreases in grey matter were also associated with decreased autonomic arousal to a social stressor (i.e., videotaped speech about an individual's faults). Similar reductions have been observed in a study of aggressive patients [8] and of pathological liars [9]. Nonetheless, such morphological and volumetric abnormalities may not necessarily relate to behaviour.

In principal, using brain imaging to look at function rather than structure should reveal stronger relationships between brain and behaviour. Using positron emission tomography scanning, neuroscientists have found attenuated resting regional cerebral blood flow in the frontal lobes of violent individuals [10] and convicted criminals [11]. In healthy volunteers, evoked anger and imagined aggressive transgressions are associated with reduced modulation of the orbital and medial PFC [12]. Collectively, these studies suggest that impulsive violent acts stem from diminished recruitment of the PFC's “inhibition” systems.

## Beyond the PFC

**Figure 1:**
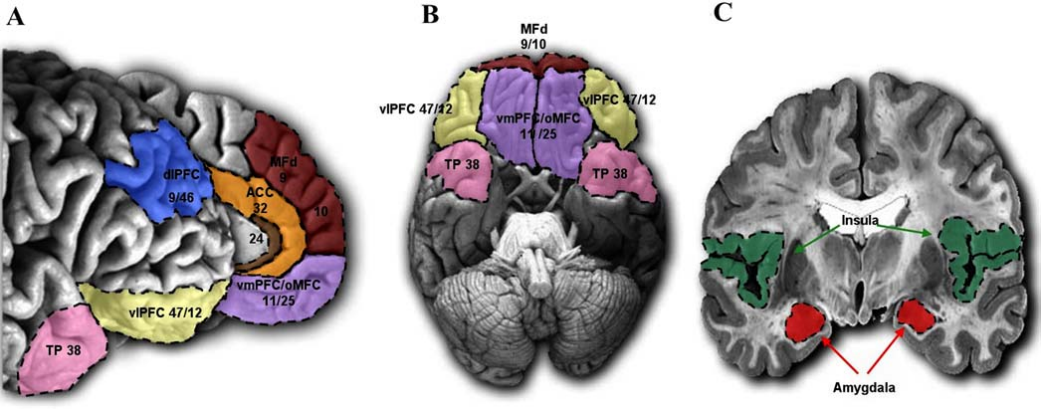
Regions Associated with Normal and Atypical Social Behaviour (A) Medial and lateral view of the PFC. (B) View of the ventral surface of the PFC and temporal poles. (C) Coronal slice illustrating the amygdalar and insular cortex. See also Table 1. ACC, anterior cingulate cortex; dlPFC, dorsolateral PFC; MFd, medial PFC; oMFC, orbitomedial PFC; TP, temporal pole; vlPFC, ventrolateral PFC; vmPFC, ventromedial PFC.

The PFC is not, however, the only area where damage may increase propensity toward behaviours deemed criminal or anti-social. It has long been known that ablation of the monkey temporal lobe, including the amygdala, results in blunted emotional responses [13] (Figure 1C). In humans, brain-imaging and lesion studies have suggested a role of the amygdala in theory of mind, aggression [14], and the ability to register fear and sadness in faces [15]. According to the violence inhibition model, both sad and fearful facial cues act as important inhibitors if we are violent towards others. In support of this model, recent investigations have shown that individuals with a history of aggressive behaviour have poorer recognition of facial expressions [16], which might be due to amygdala dysfunction [17]. Others have recently demonstrated how the low expression of X-linked monoamine oxidase A (MAOA)—which is an important enzyme in the catabolism of monoamines, most notably serotonin (5-HT), and has been associated with an increased propensity towards reactive violence in abused children [18]—is associated with volume changes and hyperactivity in the amygdala [19].

The amygdala has been a major focus of attempts to understand the poor empathy and fear responses observed in psychopathic criminals. Using functional magnetic resonance imaging (fMRI), Birbaumer and coworkers [20] presented individuals with a paradigm in which the appearance of a face on a screen was followed by a painful shock in one condition but not in a second condition. Analysis showed normal volunteers to have increased activity in the amygdala (see Figure 1) in response to faces associated with shock, whereas psychopathic individuals showed no significant change in activity in this region. In addition, psychopaths also failed to show normal increases in skin conductance responses. Importantly, Birbaumer et al.'s findings are supported by studies showing that the limbic structures (i.e., amygdala and hippocampus) are functionally abnormal in psychopathic criminals during emotional memory [21] and by studies showing how activity in the amygdala decreases with increased scores on the Psychopathy Personality Inventory [12,22]. A prevailing hypothesis is that in psychopathic criminals the prefrontal–amygdala connections are disrupted, leading to deficits in contextual fear conditioning [23], regret [24], guilt [25], and affect regulation [26].

## Does the Crime Fit the Brain?

While many behaviours can be unambiguously defined, labelling a behaviour as “criminal” is to define how the behaviour will be considered socially. That is, the very same behaviour that might not be deemed criminal in one social context (say, shooting a gun at a target at a shooting range) may be deemed criminal in another (such as shooting a gun in the direction of a crowd of people). Such definitional ambiguities are at their least frequent, however, with respect to interpersonal violence, which is broadly proscribed.

It is clear in at least some contexts that different violent anti-social behaviours can arise from different etiologies. Animal studies have shown that distinct networks underlie different types of aggression (e.g., predatory attack and defensive rage [27]). From these studies, one might expect that in humans, distinct neural topographies exist in, for example, the sexual criminal, the sadistic murderer, and the political terrorist. At first glance, such reasoning looks like phrenological folly; however, evidence does suggest that violent behaviour can be placed into two broad, yet distinct, categories: affective aggression (i.e., impulsive, autonomic arousing, and emotional) and predatory aggression (i.e., premeditated, goal-directed, and emotionless) [28].

With this dichotomy in mind, Raine and colleagues [29] reanalyzed positron emission tomography data to tease apart functional differences between premeditated psychopaths and impulsive affective murderers. Compared to controls, the impulsive murderers had reduced activation in the bilateral PFC, while activity in the limbic structures was enhanced. Conversely, the predatory psychopaths had relatively normal prefrontal functioning, but increased right subcortical activity, which included the amygdala and hippocampus. These results suggest that predatory psychopaths are able to regulate their impulses, in contrast to impulsive murderers, who lack the prefrontal “inhibitory” machinery that stop them from committing violent transgressions. Although more work is necessary, these studies strongly suggest that some kinds of criminal behaviour are associated with dysfunction of different regions of the brain.

## Does Some Criminal Behaviour Result from Mental Disorder?

A great deal of empirical research demonstrates that mental illness is higher in incarcerated populations and estimates that as many as 25% of defendants evaluated for competency are medically and legally incompetent to stand trial [30]. Moreover, only 36% of the public perceive recidivistic crime as an organic disorder [31]. Consequently, weighing discrepancies between intuitions, expert views, and empirical findings is of fundamental importance to a legal system.

Both the *Diagnostic and Statistical Manual of Mental Disorders* and *International Classification of Diseases 10* classifications of mental and behavioural disorders include APD, which is defined respectively in the two classifications as a lack of regard for the feelings of others and a failure to abide by society's rules. While it can be said that any given population of incarcerated criminals may not be a representative sample of all criminals, or even of all criminals who pass through the prison system, a systematic review of studies examining mental illness in 23,000 prisoners showed that these prisoners were several times more likely to have some form of psychosis or major depression, and ten times more likely to exhibit APD than the general population [32]. The authors suggest that, worldwide, several million prisoners have serious mental illness [32]. Several studies also show levels of head injury to be higher in violent and death-row criminals [33], while birth complications, which can often result in neurological damage (e.g., hypoxic-ischemic encephalopathy) and parental mental illness, are higher in anti-social populations [31]. More often than not, people with APD and violent behaviour have a history of childhood maltreatment or trauma [34]; having such a history has been linked to anomalous development of regions associated with anti-social behaviour, including the PFC, hippocampus, amygdala, corpus callosum, and hypothalamic–pituitary–adrenal axis [35]. Early damage to the orbitofrontal cortex in particular appears to result in poor acquisition of moral and social rules [36], thus showing the importance of the interaction between environment and brain development.

Discussing the possibility of meaningful links between some antisocial and violent behaviour and various brain disorders can, however, enrage retributivists, who point out that moral responsibility lies in the social rules by which acts are judged—not in the brain itself [37]. Nonetheless, there are many instances where brain disease can lead to antisocial behaviour, and these inevitably pose important complications for moral and legal systems that tend to divide responsibility for actions into dichotomous alternatives—guilty versus not guilty—instead of seeing responsibility as existing along a continuum. For example, compared to the general population, individuals with frontotemporal dementia, Huntington disease, and attention deficit/hyperactive disorder have a higher prevalence of episodic aggression or anti-social conduct. One disturbing example cited by Goldberg [38] is the case of a New York surgeon who, after finishing surgery, carved his signature in the patient's stomach. The surgeon was later diagnosed with Pick disease (a form of dementia associated with personality changes that presumably result from progressive degeneration of frontal and anterior temporal cortices). He was not considered responsible for his actions by experts, the jury, or even the victim. Beyond these examples lies the possibility that some forms of anti-social or violent behaviour are of unspecified origin, which could place them in the same category as many other neuropsychiatric disorders. Presumably, such unrecognized brain abnormalities might cause acts of gratuitous violence, but the individuals concerned would be considered to be criminally responsible.

To be clear, there is at present no reason to believe that all criminal behaviours, or indeed even all violent criminal behaviours, are the result of organically dysfunctional brains. However, there is ample evidence to suggest that some kinds of dysfunction are likely to increase the probability of some kinds of behaviours that society labels as criminal. This suggests that research is urgently needed to elucidate the links between mental illness, neurological disorder, and criminal conduct. And modern and rapidly improving brain-imaging techniques may contribute significantly.

## Possible Legal Implications

Advances in neuroscience could have several implications for the legal system. At the broadest level, these include (i) understanding how cognitive processes of key legal participants (such as judges and jurors) influence trial outcomes, (ii) discovering whether various assumptions underlying the evidentiary rules (such as one suggesting that “excited utterances” are less likely than average to be falsehoods) have any basis in fact, (iii) learning more about how people determine “just” punishments, (iv) anticipating how jurors may over-react to certain kinds of character evidence, (v) determining the extent of injuries from accidents, (vi) improving our abilities to detect mental biases and prejudices that may affect the proper function of legal fact-finding and decision-making, and (vii) learning more about the limits of witness memories. Yet even against this broad background, few implications for the legal system are more important than trying to gain a better understanding of important influences on criminal behaviour.

However, that very significance brings its own important challenges. On one hand, a better understanding may lead to more effective deterrence, to more effective treatment, and to more just and morally sound sentencing. On the other hand, determining criminal responsibility is a normative legal conclusion, not an empirical factual one, made in the context of a variety of often conflicting aspirations [39]. Therefore, even the best neuroscientific study can only afford factual evidence to be weighed alongside other behavioural evidence and normative considerations, rather than actually resolve the legal question as to which the factual evidence is relevant.

Generally speaking, in the Anglo-American criminal justice system, a person can be held criminally responsible if he performs a prohibited act intentionally and with a statutorily specified mental state (which may span such things as “purpose”, “knowledge”, “recklessness”, or “negligence”) [39]. Yet even if these criteria are satisfied, the defendant can be excused from liability if legally insane. That is, he may have intentionally and knowingly committed a proscribed act, but be found not blameworthy nonetheless, because a mental condition meeting a specified legal (as distinct from medical) threshold prevented him either from knowing the nature and quality of his act, or from understanding the wrongfulness of the act [40].

The possibility of being “not guilty by reason of insanity” can be traced back to the well-known M'Naghten case in 1843. While attempting to kill the British Prime Minister, Daniel M'Naghten mistakenly killed the Prime Minister's secretary. Experts maintained that M'Naghten exhibited such a vast deterioration in his reasoning abilities (believing the Prime Minister to be heading a murderous conspiracy) that he had no comprehension of the act he committed. The modern standards for determining legal insanity, in the long wake of M'Naghten, vary markedly across jurisdictions, with results that have prompted many calls for reform. For example, psychiatrists have been plagued by the need to answer dichotomously whether a defendant is “mad” or “bad” or to opine that “it is not him, it is his disease” [41]. Furthermore, medical research indicates that patients with selective damage to the PFC can often know right from wrong, but still be unable to act on such knowledge. This has naturally led defence attorneys and prosecutors to pursue more objective ways of determining whether a defendant is competent to stand trial, and if so, whether he can be held legally responsible for his actions. This, in turn, has generated significant interest in brain-imaging evidence concerning a defendant's mental functioning (Box 2).

**Box 2. Brain Fingerprinting and Lie Detection**

Lie detection technology is one of the most obvious legal uses of brain imaging, and several new companies (e.g., No Lie MRI) are beginning to commercialise their services to lawyers and prosecutors. However, despite there being several published empirical studies on lie detection, results seem to be far from conclusive. Early brain-imaging studies of how the brain responds when we willfully lie showed that specific zones of the PFC increase in activity when individuals lie—the same regions known to come online when tasks become more difficult and when we need to control or inhibit responses [67]. However, one problem with most studies of lie detection is that they use group averages, which make firm conclusions about individual cases impossible. Although more work is needed, recent studies on single individuals have shown promise, with lie detection accuracy in the range of 80%–90% [68]. Proponents argue that the use of brain imaging to detect deception is less prone to countermeasures, making it more reliable than the polygraph test [69]. Not surprisingly, government institutions have become increasingly interested (e.g., US Department of Defense) and have been criticized as being “Orwellian”. However like the polygraph, brain imaging is unlikely to be universally admissible in court until it is shown to be valid, reliable, and relevant.

Another technique—brain fingerprinting—uses electroencephalography to examine the memory and encoding related multifaceted electroencephalographic response (MERMER). To measure this, an individual is shown crime scene pictures (i.e., the murder weapon), and changes in brain activity (specifically the P300 component) are monitored. The brain reacts differently to images it recognises versus ones that it does not recognise, so, for example, if an individual did use a specific weapon to kill a person, the brain will react differently to images of the murder weapon than to images of other weapons not used in the crime. Brain fingerprinting evidence has been admitted in some cases, such as in the Iowa murder trial of Terry Harrington. However, despite its claimed potential, brain fingerprinting has been criticised for problems with developing adequate test stimuli, vulnerability to countermeasures, and—because it's patented—a failure to be appropriately verified by peer review [70].

**Figure 2:**
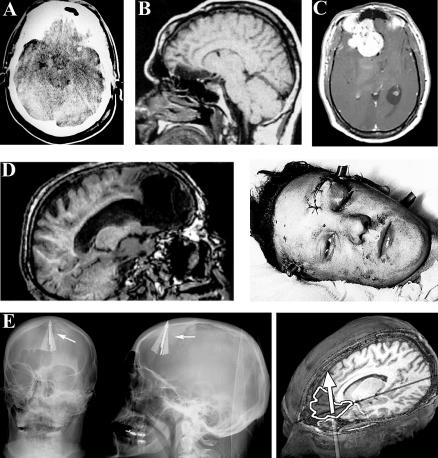
Cases Where Brain Anomalies Have, or Have Not, Been Linked to Anti-Social Behaviour (A) Brain scan of patient J. S., who exhibited sociopathic behaviour [5]. The image shows a lesion in the orbital frontal cortex. (B) fMRI sagittal slice of the brain of patient J. Z., showing a lesion that was caused by the resection of pituitary tumour [4]. This lesion led to anti-social conduct, which was not exhibited before the surgery. (C) Orbitofrontal damage associated with symptoms of paedophilia and sexual misconduct in the case of a 40-year-old male patient. (D) Photograph of a patient after head injury (right) and fMRI scan 60 years later showing PFC damage (left) [53]. This patient showed personality changes, but no signs of anti-social conduct. (E) Cranial X-ray of a man who attempted suicide with a crossbow. Although the individual exhibited premorbid APD, the PFC damage caused by the crossbow arrow resulted in reversal of anti-social conduct [54].

Several examples illustrate the kinds of contexts in which many believe that brain imaging may aid the law's ability to accurately assess a defendant's mental functioning. Consider the case of a 40-year-old man who inexplicably became a sexual impulsive with paedophilia. The patient had no prior history of sexual misconduct, but it was soon noted that he was frequenting prostitutes and that he attempted to molest his 12-year-old step-daughter. He was quickly reported to the local authorities, was found guilty of child molestation, and was sentenced to either attend a 12-step sexual addiction program or face jail. Despite a strong yearning not to go to prison, the patient could not inhibit his sexual impulses. It was soon discovered that the defendant had a large tumour pressing on his right orbitofrontal cortex (Figure 2). Upon the resection of the tumour, the patient's sexual impulsiveness diminished. When the sexual impulsiveness later reappeared, a brain scan revealed that the tumour had grown back. A second resection of tumour again diminished the patient's sexual impulsiveness [42]. Another illustration is the 1998 case of 15-year-old Kip Kinkel, who shot and killed his parents and two high-school colleagues in the state of Oregon. Brain imaging was used as evidence in court to support Kinkel's “not guilty by reason of insanity” plea. The trial defence provided evidence of small cavities in Kinkel's frontal lobe. Although there was no evidence that this abnormality caused his behaviour (Kinkel was ultimately convicted as an adult and sentenced to 111 years in prison [43]), future developments in neuroscience may again aid courts in these kinds of inquiries.

These examples raise important questions not only about the extent to which neuroimaging may affect particular trial outcomes, but also about the ways in which the legal system can come to understand changing views of the brain, assess when those views are relevant, and determine how, in appropriate circumstances, to integrate that knowledge into legal decision-making [44] (see Boxes 2 and 3). For example, recent evidence suggests that the PFC continues to mature until the age of 25 [45] and that this maturation correlates with ability in counterfactual (if–then) thinking [46]. An underdeveloped ventrolateral PFC can be directly associated with poorer cognitive control [47], which some consider a core variable in criminal activity [48]. Such research and theory likely warrants serious consideration, given the robust relationship between age and violent criminal offences. For example, British Crime Survey statistics show that individuals between the ages of 16 and 24 commit more violent acts than all other age groups combined.

**Box 3. Plausible Uses of Brain Imaging and Questions for Future Research**

Questions for which brain imaging might provide useful answers:

- Does the defendant exhibit any neurological damage?
- Do the brain abnormalities fit with the nature of the crime?
- Is the defendant faking an illness?
- Is the defendant lying about the crime?
- What is the likelihood of future transgressions?

To begin to answer such questions, society needs the following:

- More neurobiological research on anti-social and criminal populations (e.g., post-mortem histology, diffusion tensor imaging, and brain morphometry).
- A better classification of the neural activity associated with different types of criminal activity.
- A set of criteria and parameters for using imaging on single individuals with and without neurological abnormalities.
- Better understanding of the effects of intrinsic and extrinsic factors on the brain (e.g., interplay between environment, development, and genetics).
- Agreed criteria concerning validity and reliability of brain images.
- Agreed procedures for presenting imaging evidence in the courtroom.

Such statistics have a special relevance in countries such as the United States where the death penalty is applied. For example, many lawyers who oppose capital punishment of juveniles hold the view that the legal system should take such neuroscientific evidence into account (e.g., the Justice for Children Project; http://moritzlaw.osu.edu/jfc) [49]. It is possible that the 2005 decision of the Supreme Court of the United States (Roper v. Simmons) that made it illegal to use capital punishment for any offender who was under the age of 18 when he committed his crime was influenced in part by evidence presented in amicus (so-called “friends of the court”) briefs, which included neuroscientific evidence [50].

## The Limits of Brain Imaging as Evidence

There are many exciting possibilities for how law and neuroscience may eventually partner—with neuroscientists discovering new things about the brain potentially relevant to law, and law asking questions that new neuroscientific research may help address. However, it is important to keep in mind a variety of limitations of brain-imaging technology. We highlight six.

First, functional brain imaging is not mind reading. Not only can it not tell us what or how a person was thinking at the time of a legally relevant act, it also cannot tell us with reliable accuracy what a person is thinking while being scanned. In this respect, brain imaging can only provide post hoc explanations [31]. The challenge of functional brain imaging has been likened to looking from an airplane window at night: when we look down from the plane we see complex patterns of lights, which we can demarcate into towns and cities and we can gaze at their connections through linking road lights. However, from the plane we achieve little understanding of the different, social, cultural, and political differences that exist in these blobs of light [51]. With respect to fMRI, this analogy is supported on a technical level, as the details of the relationships between metabolic demand and increased neuronal activity are poorly understood.

Second, as important as brain functioning is, brain imaging provides only one window of many into the multiple influences on behaviour that can be relevant to understanding why a person acted in an anti-social manner. Such influences include the intricate interaction between genetic, prenatal, endocrinological, social, cultural, and economic factors; “No pixel in a brain will ever be able to show culpability or nonculpability” ([52], p. 100).

Third, despite showing remarkable consistency with lesion, animal model, and electrophysiological data, brain imaging is not yet in Kuhnian terms a “pure science”. Interpretation of brain scans is admittedly somewhat subjective. Anatomical landmarks in the form of gyri and sulci differ very much from individual to individual, and even in adulthood the brain is not fixed, but shows plasticity and change in response to injury that also varies from individual to individual. Moreover, in the case of fMRI, differences in haemodynamic response may not necessarily relate to neuropathology, but to vascular and endocrinological pathology. Thus, even if brain abnormalities are found, individual differences in the extent and location of the injury, and in recovery and plasticity, present major problems for the interpretation of brain images in the legal setting.

While these problems can be reduced in research through averaging across many individuals, these are critical issues when examining a single individual. For example, all the brain-imaging studies conducted on violent and anti-social populations have studied group effects. Moreover, most studies have examined adult males, and the results cannot be generalised to females and children. Accordingly, if brain imaging is to be applied to the forensic evaluation of the single patient, a standardized set of tests, procedures, and imaging parameters are needed to achieve more valid conclusions (see Box 3).

Fourth, correlations between brain function and criminal behaviour are imperfect, calling into question both the diagnostic and predictive validity of brain-imaging evidence. That is, brain defects are not observed in all violent criminals, and conversely, not all people with PFC damage exhibit anti-social behaviour. For example, one longitudinal case study showed PFC damage to result in personality changes, but without signs of anti-social behaviour [53]. Some studies have shown how prefrontal damage can even decrease anti-social behaviour [54]. Differences in the PFC may also be caused by other variables, including levels of education and alcoholism [55]. A similar pattern emerges for the amygdala, where damage can result in increased or decreased aggression [23,56]. Moreover, in court proceedings, many experts have argued against the use of ambitious speculations concerning the brain (e.g., State of Tennessee v. Paul Dennis Reid Jr., 2002, No. 38887), particularly where the link between the criminal act and the neurological damage is based solely on brain-imaging data.

Fifth, just as it would be inappropriate to expect full localization of criminality genetically [57], it would be inappropriate to expect full localization of criminality neurologically [37]. Indeed, sociologists have long provided explanations for crime and deviance without the slightest reference to the brain.

Sixth, brain images are not only powerful, they can potentially be too powerful—an effect we have referred to as the “Christmas tree phenomenon”. For example, in much the same way that a prosecutor may sway jurors with sympathetic pictures of the innocent victim, the defence may show brightly coloured images of the perpetrator's allegedly dysfunctional brain. The vividness and technological sophistication of the images may be over-weighted by the jurors, which can warp justice just as surely as can under-weighting of relevant evidence. Brain imaging can be admissible in courts of different jurisdictions (e.g., under the Federal Rules of Evidence in the United States). However, given the increasing public interest in brain imaging [58] and the misinterpretations of what brain imaging is and can do [59], it is crucial for proper legal decision-making that judges and jurors understand the limitations of brain imaging.

## Concluding Remarks

The goals of science and of law are different. However, important legal questions such as moral blameworthiness, culpability, responsibility, and the likelihood of recidivism depend to some degree on improved understandings of human behaviour. Therefore, biological advances in understanding human brain architecture and function may overlap in important ways with legal inquiries. New studies of the criminal brain are likely to shape moral views on responsibility and free will, with possible impacts on how legal systems punish and treat criminals [60].

A growing body of research gives us good reason to believe that some kinds of brain dysfunction can affect the probability of different kinds of criminal behaviours. However, despite our growing knowledge of the brain abnormalities associated with anti-social and psychopathic behaviour, there are as yet no concrete biological markers—genetic or physiological—that can predict such behaviours. Violent and anti-social behaviours undoubtedly arise from a symphony of factors. Optimal understanding will require cooperation among many disciplines such as economics, sociology, psychology, evolutionary biology, cellular physiology, and neuroscience [61].

## Abbreviations

APD: anti-social personality disorder
fMRI: functional magnetic resonance imaging
PFC: prefrontal cortex
